# Case Report: Occupationally Related Recurrent Varicella (Chickenpox) in a Hospital Nurse

**DOI:** 10.1289/ehp.7766

**Published:** 2005-06-15

**Authors:** Chih-Hung Ku, Yu-Tien Liu, David C. Christiani

**Affiliations:** 1 School of Public Health, National Defense Medical Center, National Defense University, Taipei, Taiwan; 2 Department of Environmental Health and Epidemiology, Harvard School of Public Health, Boston, Massachusetts, USA; 3 Department of Microbiology and Immunology, National Defense Medical Center, National Defense University, Taipei, Taiwan; 4 Department of Medicine, Harvard Medical School, Massachusetts General Hospital, Boston, Massachusetts, USA

**Keywords:** chickenpox, indirect fluroscent antibody, occupational exposure, polymerase chain reaction, shingles, Taiwan, varicella-zoster virus

## Abstract

Commonly accepted outcomes of varicella-zoster virus (VZV) infections include chickenpox (primary) and shingles (recurrence or latency), as well lifetime immunity against chickenpox. We report the case of a registered nurse who worked in a neurologic surgery ward in a general hospital in Taipei, Taiwan. While working there for approximately 1 year, she developed recurrent chickenpox after caring for a paraparesis patient, who had herpes zoster during hospitalization in August 2002. The varicella incubation period was 10 days, which matched the range (10–21 days). Recently negative specific serum IgM and positive specific serum IgG indicated a past VZV infection. The nurse did not get herpes zoster from the second episode of varicella on 9 August 2002 to 4 April 2005 and is now convalescing. We conclude that occupational VZV hazards exist in the health care environment and suggest testing for VZV antibody and a VZV vaccination program for susceptible health care workers.

Varicella (chickenpox), a common contagious disease of childhood, is caused by the varicella zoster virus (VZV) [Centers for Disease Control and Prevention (CDC) 2005]. VZV is characteristic of the alpha herpes viruses and establishes latency in the cells of the dorsal root ganglia after primary infection (Arvin 1996). The etiology of varicella and herpes zoster was first reported by von Bo′kay in 1888 from the observation that susceptible children often developed varicella after exposure to adults with herpes zoster (Arvin 1996; CDC 2005; von Bo′kay 1909). Varicella results from the primary VZV infection, whereas herpes zoster (shingles) is the result of reactivation (Arvin 1996; CDC 2005; Jumaan et al. 2002). Primary varicella infection usually results in lifetime immunity (CDC 2005), and second episodes of varicella are uncommon (CDC 2005; Gershon et al. 1984), but they may occur (CDC 2005). VZV disease history always indicates that varicella is the primary infection, and herpes zoster is a recurrence of the disease (Arvin 1996; CDC 1996, 1997 CDC 2005; Gershon et al. 1984; Jumaan et al. 2002), as well-documented second episodes of varicella are rare (Gershon et al. 1984). Here we report a case of apparent VZV reinfection with recurrent varicella infection in a nurse in a teaching general hospital in Taiwan.

## Case Presentation

A 25-year-old nurse, who had childhood chickenpox, was diagnosed with varicella without mention of complication [*International Classification of Diseases, Revision 9* (ICD-9) code 052.9; World Health Organization (WHO) 2001] by a dermatologic physician in a teaching general hospital after she cared for a 62-year-old male paraparesis patient who developed herpes zoster during hospitalization. She graduated from nursing school in July 2001, passed the licensing board, and then started to work in the neurologic surgery ward of a general teaching hospital in Taipei, Taiwan.

Toward the end of June, we conducted a study of occupational VZV hazards to health care workers in this hospital. The nurse was one of the volunteers who carried an air sampler for several hours in rotation with her colleagues on 9 July 2002. Saliva was collected simultaneously. Nested polymerase chain reaction (PCR) VZV DNA results were negative both in the personal air samples and in saliva.

On 13 July 2002, a 62-year-old man was sent to the emergency room due to paraparesis after he received Chinese traditional chiropractic treatment from a nonprofessional. He was diagnosed with spondylitis with a T8 compression fracture and T9 myelopathy, suspected tuberculosis (TB) of the spine, and paraparesis and was transferred to a neurologic (internal medicine) ward in the evening. The next day he underwent surgery for total laminectomy (from T8 to T9) and hook system (from T6 to T11), and was sent to the surgical intensive care unit (SICU) for 5 days. On 18 July 2002, the patient was transferred to the neurologic surgery ward where the nurse worked.

Because the patient was suspected of having TB of the spine, he underwent pleural and video-assisted thoracoscopic surgery (VATS) biopsy on 1 August. According to the nurse’s observation, the patient was worried, anxious, and under a great deal of stress regarding his health because his income was the primary financial resource of his family. On 3 August, multiple pruritic rash and vesicles were found over the patient’s abdomen and lower back flank area. Herpes zoster was confirmed by a dermatologic physician.

During the patient’s hospitalization in the neurologic surgery ward, the nurse attended the patient for several days (on 19–22 July, 29 July, 31 July–1 August, and 4–5 August 2002) before she resigned from the job on 12 August. Her regular nursing care tasks included measuring body temperature, blood pressure, and pulse; administering medicine; asking how the patient was feeling; and helping the patient to change his bed rest position.

At the age of 5 years, the nurse had been infected with chickenpox by her kindergarten-age sister, who herself was previously infected by her kindergarten classmates. At that time, the nurse, her sister, and her brother had multiple chickenpox vesicles on their faces simultaneously. Because of her previous chickenpox history, it was supposed that the nurse was immune to the VZV; while caring for the patient, she did not wear gloves, a mask, or an isolation gown before the herpes zoster was confirmed.

Because she had passed the admission examination for graduate study in a medical school, the nurse planned to resign her job to become a full-time student by 15 August. Before she resigned, she took a short vacation to her hometown in Ping-Tung County, in the south of Taiwan, on 6 August. However, on 8 August, she developed a high fever (39.5°C, 103.1°F), malaise, and headache. She went to a private clinic and was diagnosed with influenza by a physician in her hometown. The next day, in addition to the prodromal symptoms, pruritic rash started to appear on her face, neck, and trunk. She came back to the teaching general hospital in Taipei on 9 August and was diagnosed with varicella without mention of complication (ICD-9 code 052.9) by another dermatologic physician (not the herpes zoster patient’s dermatologic physician). The nurse received treatment with Allegra (fexofenadine, 60 mg twice per day; Hoechst, Kansas City, MO, USA), chlorpheniramine (4 mg three time per day; Mine Ta Chemistry Pharmacy Co., Ltd., Tai Chung, Taiwan), and Scanol (acetominophen, 500 mg three times per day; Scanpharm, Birkerød, Denmark) for 14 days. Because her clinical symptoms were very clear, no laboratory confirmation testing was performed at that time. However, to confirm this second varicella episode before reporting this case, we invited the nurse to take serologic tests for varicella antibody on 4 April 2005. The results indicated that VZV-specific IgM was negative [< 1:20; Merifluor VZV IgM indirect fluorescent antibody, (IFA); Meridian Bioscience Inc., Cincinnati, OH, USA], but the VZV IgG was positive (1:80 on the basis of > 1:10, Merifluor VZV IgG IFA), with high sensitivity (93.9% for IgM and 100% for IgG) and high specificity (100% for both antibodies) according to the manufacturer’s instructions.

Negative specific serum IgM and positive specific serum IgG indicated a past VZV infection. Because varicella and herpes zoster both resulted from the same antigen, the VZV IgG-positive reaction was excluded from the herpes zoster. It was also excluded because the nurse did not get herpes zoster from the second episode of varicella on 9 August 2002 to 4 April 2005. The nurse is now convalescing.

## Discussion

The nurse was diagnosed with an acute chickenpox infection by a dermatologic physician at the teaching general hospital on the basis of her clinical symptoms and signs, including a high fever (39.5°C), malaise, and headache prior to the rash appearing on her face, neck, and trunk. The duration of the high fever and rash were 5 and 14 days, respectively. Fever and malaise may appear 1–2 days before rash onset in chickenpox primary infections in adults, whereas rash is usually the first sign of the disease in children (CDC 2005). According to the CDC case classification (Jumaan et al. 2002), a confirmed case is defined as one that is confirmed by laboratory testing or that meets the clinical case definition and is epidemiologically linked to a confirmed or a probable case (Jumaan et al. 2002). Recently, negative specific serum IgM and positive specific serum IgG indicated a past VZV infection. The nurse did not get herpes zoster from the second episode of varicella on 9 August 2002 to 4 April 2005. Our case matches both definitions because the vesicular illness preceded acute onset with diffuse maculopapulovesicular rash and without other apparent cause (Jumaan et al. 2002), and it was epidemiologically linked to the care of a confirmed herpes zoster patient.

The air sampling study was performed on 9 July 2002 in the neurologic surgery ward, and the paraparesis patient was admitted to the hospital on 13 July 2002 and transferred to the neurologic surgery ward on 18 July 2002. The patient’s rash was first reported on 3 August 2002. It is not surprising that the air sampling result was negative. In addition, it is unlikely that the nurse was infected from the air sampling equipment because the assembled cassettes and sampling pump with tubing were put in a laminar flow unit with ultraviolet light for 8 hr before sampling, and sampled cassettes were replaced with a new assembled cassette every day.

To investigate this uncommon case, we considered four parameters: the agent (VZV), infectious routes, the host (the nurse), and the hospital environment (hospital ward).

### Agent.

Previous reports have mentioned the same etiology of varicella and herpes zoster (Arvin 1996; CDC 2005; von Bo′kay 1909). In 1925, children without varicella history developed varicella after being inoculated with fluid recovered from the herpes zoster lesions (infectious virus), which demonstrated the transmissibility of the agent (Arvin 1996; Kundratitz 1925). In the present case, the nurse developed a recurrence of varicella after nursing a herpes zoster patient, although she had been infected by her sister when she was 5 years of age.

Varicella has been reported as the disease most commonly confused with smallpox (Jumaan et al. 2002); however, smallpox was eradicated from Taiwan in 1955 (Center for Disease Control, Taiwan 2004a, 2004b), and the routine smallpox vaccination program in Taiwan ended in 1979 after the disease was eradicated worldwide following the WHO campaign (BiotechEast 2003; Center for Disease Control, Taiwan 2004a, 2004b; Lee 2003). Because the nurse was born in 1978, it is extremely unlikely that her rash at 5 years of age was smallpox.

New variants of VZV have been reported recently, including VZV-MSP, isolated in St. Paul–Minneapolis, MN (Santos et al. 1998, 2000), and VZV-BC, isolated in British Columbia (Tipples et al. 2002). We do not know whether there is a mutant strain of VZV in Taiwan; thus this is a good index case for further study. Another consideration is that the nurse’s immunity was insufficient against the second VZV attack, since she was infected initially over 20 years earlier by her sister. Unfortunately, it was not possible to check her VZV antibody titers before her recurrent varicella episode.

### Infection routes and incubation period.

It has been documented that cases of herpes zoster are the infectious sources of chickenpox in susceptible persons (Arvin 1996; CDC 2005). VZV is transmissible by respiratory routes (Arvin 1996; CDC 2005). Possible infection routes while the nurse was caring for the patient included communicating with the patient and helping the patient change his bed rest position without employing protection such as a mask or gloves.

Previous PCR research showed that VZV is transmissible for 24–48 hr before rash onset, which is consistent with epidemiologic evidence (CDC 2005; Koropchak et al. 1991), or 4–5 days after a rash appears (CDC 2005).

The chickenpox incubation period is from 14 to 16 days after exposure (Arvin 1996; CDC 2005), with a range of 10–21 days (CDC 2005). In this case, the paraparesis patient had the rash on 3 August 2002. The nurse had cared for him on 31 July and on 1, 4, and 5 August. The most likely infection dates of this episode are 31 July and 1 August, since the rash appeared on the nurse’s face, neck, and trunk on 9 August, which is within the range of 10 days. In addition, recent VZV-IgG serologic tests indicated that the antibody titer is still high (1:80 on the basis of 1:10 of a positive criterion). This method was simple and highly sensitive and allowed for the rapid and reliable determination of immunity to VZV (Sauerbrei et al. 2004). Because varicella and herpes zoster resulted from the same antigen, VZV, the VZV IgG-positive reaction was excluded from herpes zoster. It was also excluded because the nurse did not get herpes zoster from the second episode of varicella on 9 August 2002 to 4 April 2005.

### Host.

In addition to a lack of protection (gloves, mask) while caring for the patient, stress may be another important host factor contributing to recurrence. The nurse reported that before she resigned from the hospital, she felt stress both from the nursing practice and the logistics of her resignation, as well as anxiety about her impending study in graduate school. In fact, during our study of occupational VZV hazards to healthcare workers in early July 2002, the nurse reported performance anxiety about graduate school and stress from multiple clinical duties and logistic matters concerning her resignation, such as the fact that she was on duty on the date of graduate school registration, 5 August 2002. According to the nurse’s own report after the recurrence, we believe that stress played an important role in this recurrent varicella.

### Environment.

Documented environmental VZV DNA included sampling from the air of the active patient’s room (Sawyer et al. 1994), or the active patient’s family, including air conditioner filter, table, television remote control, and door handle (Asano et al. 1999). In our study, 20.5% of the 44 air samples from different departments of the same hospital were VZV DNA positive. VZV is highly temperature sensitive (inactivated at 56–60°C) in the environment and is not infectious if the virion’s envelope is disrupted (Arvin 1996). The temperature in the hospital was controlled by the central operation department at about 25°C.

The nurse was a young and unmarried female, with only one year of work history. Her disease appears to be recurrent varicella, which is very uncommon, and it appears to be occupationally related. Documented VZV infection may cause significant morbidity or mortality, affecting the mother, the fetus, or the newborn (Arvin 1996; CDC 1996, 1997 CDC 2005; Harger et al. 2002; Jumaan et al. 2002), as well increasing the risk of premature delivery when infected during late pregnancy (Arvin 1996; Paryani and Arvin 1986). We report this case to emphasize that a varicella infection history may not be sufficient for determination of VZV immunity, contrary to the common belief that a reliable history of varicella is a valid measure of immunity because the rash is distinctive and subclinical cases rarely occur (CDC 1996, 2005; Jumaan et al. 2002). We think that VZV antibody testing may be necessary for health care workers, especially for new female workers.

Documented methods of VZV antibody detection include complement fixation, IFA, fluorescent antibody to membrane antigen (FAMA), neutralization, indirect hemagglutination, immune adherence hemagglutination, radioimmunoassay, latex agglutination, and enzyme-linked immunosorbent assay (CDC 1996). Although IFA, FAMA, neutrilization, and radioimmunoassay have been reported to be sensitive but time consuming (CDC 1996), a highly sensitive, specific, and rapid IFA test using VZV-infected A549 cells as antigen has been developed (Sauerbrei et al. 2004). The sensitivity and the specificity of this method are 100%, compared to the FAMA test, with the lowest limit of detection 50 mIU/mL versus 250 mIU/mL anti-VZV IgG for IFA and FAMA, respectively (Sauerbrei et al. 2004).

## Conclusion

Occupational VZV hazards exist in the health care environment. The traditional concept of VZV lifetime immunity after the primary varicella infection may not be appropriate in a health care setting. We suggest checking serologic titers for VZV antibody, followed by a VZV vaccination for nonimmune health care workers.
